# Wide‐Bandgap Rare‐Earth Iodate Single Crystals for Superior X‐Ray Detection and Imaging

**DOI:** 10.1002/advs.202206833

**Published:** 2023-03-22

**Authors:** Xieming Xu, Fang Wang, Weiwei Xu, Hao Lu, Lingfei Lv, Hongyuan Sha, Xiaoming Jiang, Shaofan Wu, Shuaihua Wang

**Affiliations:** ^1^ Key Laboratory of Optoelectronic Materials Chemistry and Physics Fujian Institute of Research on the Structure of Matter Chinese Academy of Sciences Fuzhou Fujian 350002 China; ^2^ University of Chinese Academy of Sciences Beijing 100049 China; ^3^ Fujian Science & Technology Innovation Laboratory for Optoelectronic Information of China Fuzhou Fujian 350002 China

**Keywords:** anisotropic response, rare‐earth iodates, wide bandgap, X‐ray detection, X‐ray imaging

## Abstract

Semiconductor‐based X‐ray detectors with low detectable thresholds become critical in medical radiography applications. However, their performance is generally limited by intrinsic defects or unresolved issues of materials, and developing a novel scintillation semiconductor for low‐dose X‐ray detection is a highly urgent objective. Herein, a high‐quality rare‐earth iodate Tm(IO_3_)_3_ single crystal grown through low‐cost solution processing is reported with a wide bandgap of 4.1 eV and a large atomic number of 53.2. The roles of I—O and Tm—O groups for charge transport in the Tm(IO_3_)_3_ are revealed with the structural difference between the [101] and $\left[\bar{1}01\right]$ crystal orientations. Based on anisotropic responses of material properties and detection performances, it is found that the [$\bar{1}01$] orientation, the path with fewer I—O groups, achieves a high resistivity of 1.02 × 10^11^ Ω cm. Consequently, a single‐crystal detector exhibits a low dark current and small baseline drifting due to the wide bandgap, high resistivity and less ion migration of Tm(IO_3_)_3_, resulting in a low detection limit of 85.2 nGy_air_ s^−1^. An excellent X‐ray imaging performance with a high sensitivity of 4406.6 µC Gy_air_^−1^ cm^−2^ is also shown in the Tm(IO_3_)_3_ device. These findings provide a new material design perspective for high‐performance X‐ray imaging applications.

## Graphical abstract

A wide‐bandgap rare‐earth iodate Tm(IO_3_)_3_ single crystal with a low detection limit (85.2 nGy_air_ s^−1^) is used for superior X‐ray detection and imaging. Structural differences and anisotropic responses of [101] and [$\bar{1}01$] crystal orientations reveal the charge transport role that I—O groups play, providing a new perspective for the material design of wide‐bandgap scintillation materials in X‐ray imaging applications.

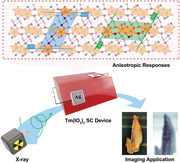

## 1 Introduction

X‐ray detectors are widely used in medical diagnosis and security inspection applications, as well as in scientific research for nuclear and high‐energy physics fields.^[^ ^1^, ^2^, ^3^, ^4^ ^]^ While most of the detectors are used for indirect‐type X‐ray detection, they require additional photoelectric conversion compared to those that use the direct detection approach.^[^ ^5^ ^]^ A direct X‐ray detector based on scintillation semiconductors is an attractive option for practical medical radiography based on current well‐established semiconductor processing techniques.^[^ ^6^ ^]^ Since reducing patient radiation dose while maintaining image quality is a key clinical need and a prevailing research trend, low dark current, small baseline drifting and robust operational stability are essential requirements for a direct X‐ray detector; these criteria are also closely related to the intrinsic material properties of the scintillation semiconductor. Numerous typical inorganic semiconductors, including Si,^[^ ^7^ ^]^ *α*‐Se,^[^ ^8^ ^]^ HgI_2_,^[^ ^9^ ^]^ CdTe^[^ ^10^ ^]^ and CdZnTe,^[^ ^11^ ^]^ have been used in direct X‐ray detection applications, but most do not perform well against the abovementioned criteria due to inherent defects or unresolved issues of materials; small bandgaps (1.12–2.25 eV)^[^ ^12^ ^]^ brings increased dark currents due to thermal noise, and materials with low atomic numbers (14–34) absorb X‐ray photons poorly. Although these issues have been addressed to some extent through material modifications^[^ ^13^, ^14^ ^]^ and device optimizations,^[^ ^15^, ^16^ ^]^ stringent growth conditions and high costs have also significantly limited the application potentials of these materials. Therefore, exploring scintillation materials that are cost‐effective and have high atomic numbers and wide bandgaps is an essential objective.

Rare‐earth iodates, which are classical inorganic salts, have been intensively investigated for their nonlinear optical properties^[^ ^17^, ^18^, ^19^ ^]^; they are also attracting attention in other areas because they are prepared using simple and low‐cost methods and are thermally stable to at least 400 °C.^[^ ^20^ ^]^ As ionic compounds with strong coulomb potentials, iodate materials typically exhibit large bandgaps (3.97–4.61 eV)^[^ ^21^, ^22^, ^23^ ^]^ that are associated with high transmittance values that avoid visible‐light interference. It should be noted that wide‐bandgap materials, such as silicon carbide^[^ ^24^ ^]^ (3.2 eV), gallium nitride^[^ ^25^ ^]^ (3.4 eV), gallium oxide^[^ ^26^, ^27^ ^]^ (4.9 eV) and diamond^[^ ^28^ ^]^ (5.5 eV), have been reported as promising candidates for use in X‐ray detection applications; they are mechanically tougher and difficult to damage by radiation than materials with lower bandgaps.^[^ ^29^ ^]^ Furthermore, the large atomic number of a rare‐earth metal ensures adequate X‐ray absorption, as demonstrated by reportedly rare‐earth iodates^[^ ^30^ ^]^ and polyiodates^[^ ^31^ ^]^ as the indirect‐type X‐ray detector. With this background in mind, we reason that wide‐bandgap rare‐earth iodates, as a new class of scintillation semiconductor, have significant potential for use in practical detection and imaging applications.

In this work, we report a thulium iodate (Tm(IO_3_)_3_) single crystal for high‐performance X‐ray detection and imaging. Sufficient X‐ray absorption is ascribable to the heavy atomic composition of Tm(IO_3_)_3_, while its wide bandgap, another essential property, produces less thermally activated carriers at room temperature, thereby fulfilling some of the strict scintillation material requirements. The observed anisotropic response is attributed to the structural difference between the [101] and [$\bar{1}01$] crystal orientations of Tm(IO_3_)_3_, which indicates material property and detection performance are affected by the group arrangement of I—O and Tm—O groups; the [$\bar{1}01$] orientation, which has less I—O groups, exhibits higher resistivity and less ion migration, resulting in a lowest detectable X‐ray dose rate of 85.2 nGy_air_ s^−1^. Moreover, in addition to the superior stability under the radiation of 507.5 Gy_air_, a vertically structured Tm(IO_3_)_3_ single‐crystal detector reaches a high sensitivity of 4406.6 µC Gy_air_^−1^cm^−2^. High‐quality and stable X‐ray imaging performances based on a single‐pixel 2D‐scan imaging system highlight the significant practical application potential of our rare‐earth iodate device.

## 2 Result and Discussions

**Figure 1:**
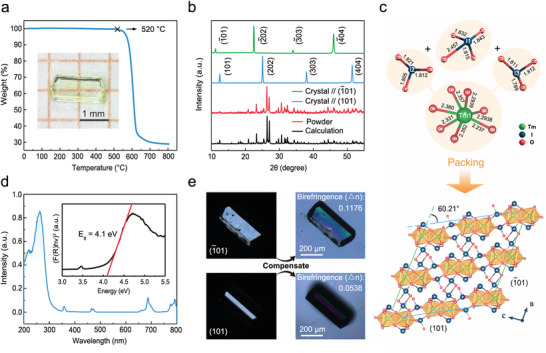
Characterizing the Tm(IO_3_)_3_ single crystal. a) Thermogravimetric analysis curve of the Tm(IO_3_)_3_ sample. Inset: photographic image of a Tm(IO_3_)_3_ single crystal. b) XRD patterns of the Tm(IO_3_)_3_ single crystal and powder. c) The coordination environment (upper) and crystal structure (lower) of Tm(IO_3_)_3_. d) UV–Vis absorption spectrum of Tm(IO_3_)_3_. Inset: optical bandgap calculated from the corresponding Tauc plot. e) Photographic images of the (101) and $\left(\bar{1}01\right)$ faces of a Tm(IO_3_)_3_ single crystal before and after compensation under an orthogonal polarizer.

An optimized slow‐cooling multi‐rate solvothermal method was used to grow Tm(IO_3_)_3_ single crystals (Figure S1, Supporting Information). A stable thermal field with programmed cooling and constant temperature sections that reduce internal stress favor the formation of a high‐quality crystal. Transparent Tm(IO_3_)_3_ single crystals ≈2.0 × 1.0 × 0.4 mm^3^ in size that contain two groups of main crystal faces (one broader and one narrower) had grown after half a month, as seen in the inset of **Figure** **1**a. Polarized optical microscopy images acquired along the main crystal faces indicate that no cracks or envelopes are seen inside the single crystal, which shows that this Tm(IO_3_)_3_ single crystal is highly optically uniform (Figure S2, Supporting Information); meanwhile, the high‐resolution X‐ray rocking curve provided a narrow full‐width at half‐maximum (0.008–0.013°), which demonstrates an ultra‐high crystallinity of our Tm(IO_3_)_3_ single crystal (Figure S3, Supporting Information). Furthermore, Tm(IO_3_)_3_ the sample was subjected to thermogravimetric analysis; the high thermal‐decomposition temperature of 520 °C and the heat‐absorption peak observed at 615 °C reflect the superior thermal stabilities of iodate materials, as shown in Figure 1a and Figure S4, Supporting Information.

Tm(IO_3_)_3_ was also analyzed by X‐ray diffraction (XRD), the results of which are shown in Figure 1b. A series of diffraction peaks with obvious preferred orientation reveals the growth habit of Tm(IO_3_)_3_ crystals with the two main crystal faces of (101) and $\left(\bar{1}01\right)$; the broader one is the $\left(\bar{1}01\right)$ face. Moreover, the diffraction peaks acquired from powder samples closely match those calculated from Tm(IO_3_)_3_ single‐crystal data (Table S1, Supporting Information). Tm(IO_3_)_3_ belongs to the monoclinic (P2_1_/n) space group, with lattice parameters *a* = 8.689(7) Å, *b* = 5.990(5) Å, *c* = 14.932(4) Å and *β* = 96.960(2)°. The band valence sum (BVS) of Tm in Tm(IO_3_)_3_ was calculated by the bond valence concept,^[^ ^32^ ^]^ which revealed a coordination number of seven based on a reasonable BVS (2.986) for the TmO_7_ polyhedron, with Tm—O distances in the 2.237(9) to 2.360(8) Å range (Tables S2 and S3, Supporting Information). Figure 1c (upper) shows that I(2) and I(3) are coordinated to three O atoms with distances between 1.789(8) and 1.822(9) Å for the trigonal pyramid of the IO_3_ group, while I(1) exists as an IO_3+1_ polyhedron (BVS = 5.042) that contains three short I—O bonds (1.815(8) to 1.843(9) Å) and one long distance (2.458(0) Å). IO_3+1_ polyhedra are prevalent in iodate compounds, with a fourth I—O band also typically formed between two iodate anions, with common distances on the order of 2.5 Å.^[^ ^33^, ^34^ ^]^ It is noteworthy that the extra O(2) atom in I(1)O_3+1_ originates from a crystallographically symmetrical transformation (Figure S5, Supporting Information). The TmO_7_ polyhedrons corner‐share with two I(1)O_3+1_, two I(2)O_3_, and three I(3)O_3_ groups (Figure S6, Supporting Information). Each I(3)O_3_ group is coordinated to three different TmO_7_ groups, and the I(2)O_3_ groups contain two O atoms coordinated to different Tm atoms and one unworked O atom. The TmO_7_ groups are mainly linked with these two I—O groups to form a Tm—I—O layer parallel to the (101) plane, and the layers are further interconnected by two bridging I(1)O_3+1_ groups to construct an iodate framework with intra‐ and inter‐ Tm—I—O layers that are clearly structurally difference (Figure 1c, lower). According to the literature, there is anisotropy of charge collection as a result of structural differences in the 1D chain^[^ ^35^ ^]^ and 2D layered structure.^[^ ^36^ ^]^ The (101) and $\left(\bar{1}01\right)$ planes, corresponding to the (101) and $\left(\bar{1}01\right)$ crystal face of Tm(IO_3_)_3_, intersect at 60.21°, and crystal orientations perpendicular to these two planes correspond to the intra‐ and interlayer directions of the iodate framework. Therefore, this structural difference enables possible anisotropic material properties and detection performances along the two main crystal faces of Tm(IO_3_)_3_ that facilitate the functional exploration in rare‐earth iodate groups.

We further examined the optical properties of Tm(IO_3_)_3_ by absorption spectroscopy in the visible‐light region (300–800 nm), which revealed an absorption of less than 15% (Figure 1d). The optical bandgap was calculated to be 4.1 eV, which is larger than those of commercially available inorganic scintillation semiconductors^[^ ^12^ ^]^: namely Si (1.12 eV), CdTe (1.5 eV), and *α*‐Se (2.25 eV) (Figure 1d, inset). A wide bandgap suppresses interference from heat and visible light to deliver a low dark current, which is critical for device performance. Additionally, Tm(IO_3_)_3_ exhibits optical anisotropy in the (101) and $\left(\bar{1}01\right)$ crystal faces (Figure 1e), with birefringence values of 0.0538@546nm and 0.1176@546nm, respectively, determined based on the interference color compensation and the calculation formula *R* = ∆*n* × *T*, where *R* is the optical path difference, *T* is the thickness and ∆*n* is the birefringence; the *T* and *R* data acquired for the two crystal faces are shown in Figure S7, Supporting Information. Moreover, the 57.89° angle measured between (101) and $\left(\bar{1}01\right)$ face is close to the theoretical angle of 60.21°. The birefringence value of Tm(IO_3_)_3_ lies within the range previously reported for iodates, including Sr[B(OH)_4_](IO_3_)^[^ ^37^ ^]^ of 0.0536@589nm, Bi_3_OF_3_(IO_3_)_4_^[^ ^38^ ^]^ of 0.057@532nm and Ba(IO_3_)F^[^ ^21^ ^]^ of 0.1253@589nm. It should be noted that anionic groups mainly contribute to birefringence performance in the previous reports.^[^ ^39^ ^]^

**Figure 2:**
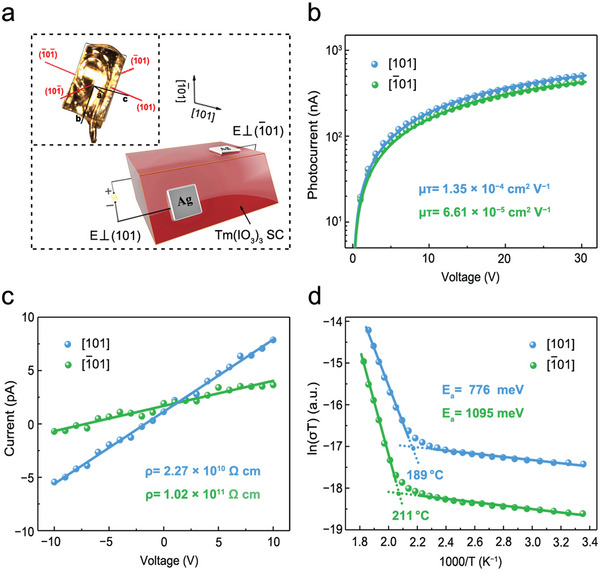
Electronic properties of the Tm(IO_3_)_3_ single crystal. a) Schematic of the vertical device prepared along the [101] and [$\bar{1}01$] crystal orientations of the Tm(IO_3_)_3_ single crystal. Inset: photographic image of the single crystal orientation. b) Bias‐dependent photoconductivities of the [101] and [$\bar{1}01$] orientation detectors. c) *I*–*V* curves of the [101] and [$\bar{1}01$] devices acquired in the dark. d) Temperature‐dependent conductivities of the [101] and [$\bar{1}01$] devices measured in the dark.

The electronic properties of Tm(IO_3_)_3_ single crystal were assessed in two crystal orientations, the results of which are displayed in **Figure** **2**. A vertical Ag/iodate/Ag structure device was prepared on two groups of parallel crystal faces to provide applied electric fields, as shown schematically in Figure 2a. Energy levels of the Tm(IO_3_)_3_ device were studied using ultraviolet photoelectron spectroscopy, as shown in Figure S8, Supporting Information. The absolute value of the electron affinity of Tm(IO_3_)_3_ is greater than that of the work function of the silver electrode, resulting in a Tm(IO_3_)_3_‐Ag ohmic contact. The crystal face indices were acquired using the single crystal orientation method, which ensured the validity of the following experiments (Figure 2a, inset). The charge‐carrier mobility‐lifetime (µ*τ*) product plays an important role in charge collection of scintillation semiconductors. The bias‐dependent photoconductivity curve along [101] and [$\bar{1}01$] orientations are clearly different (Figure 2b); the µ*τ* products were determined by fitting to be 1.35 × 10^−4^ and 6.61 × 10^−5^ cm^2^ V^−1^, respectively, which indicates that the [101] orientation is better able to collect charge. Notably, the fitted µ*τ* product method, which is based on the steady‐state photoconductivity and a modified Hecht equation, is commonly applied to reported scintillation materials^[^ ^40^ ^]^; the µ*τ* product for Tm(IO_3_)_3_ single crystal is comparable to those of commercial materials, as shown in Table S4, Supporting Information. The two orientations exhibited similarly behaved relative dielectric constants, which were determined to be 223.4 and 71.7; these values increased minimally (6%) when exposed to light due to the large bandgap of the Tm(IO_3_)_3_ single crystal (Figure S9, Supporting Information).

The resistivity of two crystal orientations reached 2.27 × 10^10^ and 1.02 × 10^11^ Ω cm, respectively, which highlights the electronic anisotropy of the Tm(IO_3_)_3_ single crystal (Figure 2c). These intrinsic resistivities (10^10^–10^11^ Ω cm) are at least one order of magnitude greater than the reported Si of 10^4^ Ω cm and CdTe of 10^8^–10^9^ Ω cm^[^ ^12^ ^]^; hence, the low dark current generated in Tm(IO_3_)_3_ is in favor of the low‐dose X‐ray detection. Figure 2d reveals that the temperature‐dependent conductivity curves show dramatic rise in the high‐temperature region; such a rise is commonly attributable to the ionic conduction resulting from thermal‐activated ion migration. The data were fitted by the Nernst–Einstein relationship^[^ ^36^ ^]^ to determine activation energies (*E_a_*) of 776 and 1095 meV for the [101] and [$\bar{1}01$] orientations, respectively, confirming that ionic conduction mostly dominates in the respective heat zones (195–270 and 215–280 °C). Furthermore, the small *E_a_* values of 33 and 36 meV for [101] and [$\bar{1}01$] orientations, respectively, in the low‐temperature regions (25–160 and 25–180 °C) indicate that the Tm(IO_3_)_3_ single‐crystal devices retain electronic conduction. Note that the conduction transition temperatures of 189 and 211 °C are far greater than reported values^[^ ^36^, ^41^ ^]^ and the actual operating temperature, which provides strong evidence for stable detection with less ion‐migration interference at room temperature; interestingly, the [$\bar{1}01$] orientation has larger resistivity and more inhibiting toward ion migration. We conclude that the anisotropic properties of Tm(IO_3_)_3_ are favorable for exploring the effects of structure during X‐ray detection.

**Figure 3:**
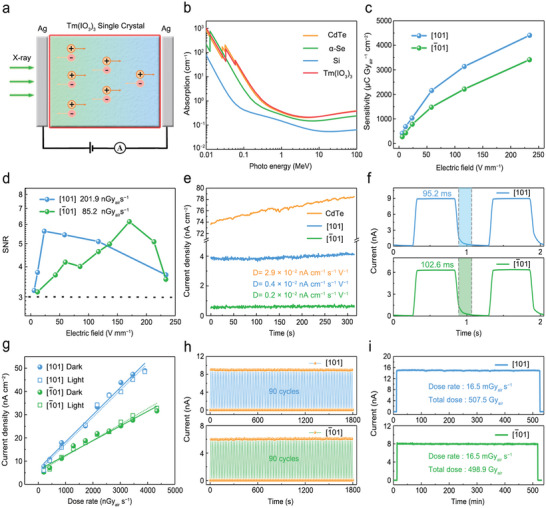
Detection performance of the Tm(IO_3_)_3_ devices. a) Schematic diagram of an X‐ray single‐crystal detector. b) X‐ray absorptions of CdTe, *α*‐Se, Si, and Tm(IO_3_)_3_ as functions of photon energy. c) X‐ray sensitivities and d) signal‐to‐noise ratios (SNRs) of the [101] and [$\bar{1}01$] orientation devices as functions of the electric field. e) Baselines of the CdTe single‐crystal device and the [101] and [$\bar{1}01$] devices as functions of time. f) Temporal X‐ray responses of the [101] and [$\bar{1}01$] devices at 233.8 V mm^−1^. g) X‐ray photocurrent densities of the [101] and [$\bar{1}01$] devices as functions of dose rate in the dark (solid lines) and when exposed to light (dotted lines). Operational stabilities of the Tm(IO_3_)_3_ devices under h) repeated on–off X‐ray radiation and i) continuous radiation.

A rare‐earth iodate X‐ray detector with a vertical device structure was fabricated using a Tm(IO_3_)_3_ single crystal to investigate the X‐ray detection performance in two crystal orientations, [101] and [$\bar{1}01$], as shown in **Figure** **3**a. Electron–hole pairs are produced inside Tm(IO_3_)_3_ single crystal following the absorption of ionizing radiation. The average energy (W) produced by the electron–hole pairs is calculated as follows: *W* = 2*E*_g_ + 1.43 eV,^[^ ^42^ ^]^ where *E*_g_ is the bandgap of the scintillation material; *W* value of Tm(IO_3_)_3_ was calculated to be 9.63 eV. The electron and hole then drift to each side of the crystal under the applied electric field and are collected by electrodes to generate a photocurrent. Additionally, the X‐ray photon energy (*E*) is exponentially attenuated by material absorption, as described by: *I*=*I*_0_exp( − *αx*), where *I*_0_ is the initial X‐ray energy intensity, *I* is the attenuated intensity, *α* is the linear absorption coefficient and *x* is the thickness of the material. Consequently, *α* is a critical scintillation materials parameter that determines its capacity to absorb radiation. Figure 3b shows that Tm(IO_3_)_3_ has a notably greater *α* value than Si over the entire energy spectrum, and is even higher than those of *α*‐Se and CdTe by energies in hundreds of kiloelectronvolts to megaelectronvolts range due to the large K absorption edge of I (33.164 keV) and Tm (59.335 keV). The attenuation efficiency of Tm(IO_3_)_3_ and commercial materials are shown in Figure S10, Supporting Information, for 50 keV X‐ray that is commonly used, as well as Tm(IO_3_)_3_ with a small thickness (<1 mm) is able to sufficiently absorb 99% of X‐ray photons. According to the relationship of *α*∝*Z* ^4^/*E* ^3^, the excellent attenuation performance of the Tm(IO_3_)_3_ rare‐earth iodate is attributed to its large effective atomic number (*Z*_eff_ = 53.2@50keV) calculated by the direct method,^[^ ^43^ ^]^ as detailed in Figure S11, Supporting Information, which facilitates effective absorption of the 50 keV X‐ray used in actual medical applications.

Sensitivity is a common figure of merit used to evaluate the device performance of a direct‐type X‐ray detector. Accordingly, the sensitivity of [101] and [$\bar{1}01$] devices were assessed in a range of electric fields (Figure 3c), with maximum values of 4406.6 and 3412.8 µC Gy_air_^−1^cm^−2^ recorded at the same electric field (233.8 V mm^−1^); sensitivities were calculated from the 9.9–50.6 µGy_air_ s^−1^ dose‐rate region in this work. The sensitivity of the Tm(IO_3_)_3_ is superior to most of the reported materials, as seen in Figure S12 and Table S5, Supporting Information; the value is at least two orders of magnitude larger than that of a typical *α*‐Se X‐ray detector^[^ ^44^ ^]^ (20 µC Gy_air_^−1^ cm^−2^). Tm(IO_3_)_3_ device performance was also further compared with a CdTe single‐crystal detector whose properties are in agreement with the literature^[^ ^12^ ^]^ (Figure S13, Supporting Information). While the CdTe device delivered a much higher current density than our devices, the sensitivity of CdTe device is only 89% of that of the [101] device, which were compared at the same electric field of 11.7 V mm^−1^ due to the uneven curve at the larger electric field (23.4 V mm^−1^), as shown in Figures S14 and S15, Supporting Information. Moreover, the device performance of Tm(IO_3_)_3_ detectors at higher energy X‐ray is exhibited in Figure S16, Supporting Information, confirming that the Tm(IO_3_)_3_ detector still works effectively at 60–120 keV_p_ regions. The positive correlation between X‐ray energy and sensitivity indicates that a stronger response to higher X‐ray energy at the same dose rate, which can be explained by an enough absorption of Tm(IO_3_)_3_ for 50–120 keV_p_ X‐ray particles (Figure S17, Supporting Information).

A low detection limit is required to ensure that a low radiation dose can maintain satisfactory X‐ray detection and imaging performance. Relationships between the signal‐to‐noise ratio (SNR) and electric fields for our two Tm(IO_3_)_3_ devices are shown in Figure 3d. A dose rate with SNR = 3 is defined as a detection limit according to the IUPAC standard. The [$\bar{1}01$] orientation device exhibited an extremely low detection limit of 85.2 nGy_air_ s^−1^, which is more than sixty times lower than the typical dose rate of 5.5 µGy_air_ s^−1^ used in medical therapy,^[^ ^45^ ^]^ and another orientation device has a larger value of 201.9 nGy_air_ s^−1^. The noise current performances are detailed in Figure S19, Supporting Information. It is noteworthy that the difference between the two orientations is largely ascribable to the anisotropic resistivities and activation energies of Tm(IO_3_)_3_ single crystal. A similar outcome was also found in the comparison between Tm(IO_3_)_3_ and CdTe devices (Figure S20, Supporting Information); the CdTe device reached a detection limit of 1.29 µGy_air_ s^−1^, which is significantly larger than Tm(IO_3_)_3_ devices at the same electric field, due to the smallest resistivity of 2.23 × 10^8^ Ω cm. Moreover, these devices were further compared by the baseline drifting measurement, which is a non‐negligible factor for low‐dose detection. In Figure 3e, the current drifting (*D*) values of a CdTe device and the [101] and [$\bar{1}01$] Tm(IO_3_)_3_ devices were calculated to be 2.9 × 10^−2^, 0.4 × 10^−2^ and 0.2 × 10^−2^ nA cm^−1^ s^−1^ V^−1^ at 23.4 V mm^−1^, respectively, based on an equation of *D* = (*J_t_* − *J*_0_)/*tE*, where *t* is the duration, *E* is the electric field, *J_t_* and *J*_0_ are the initial and final current densities. Please note that the excellent performance for suppressing dark currents in [$\bar{1}01$] orientation of Tm(IO_3_)_3_ was confirmed by its lowest values in baseline drifting (0.2 × 10^−2^ nA cm^−1^ s^−1^ V^−1^) and dark current density (0.6 nA cm^−2^).

Temporal response and operational stability experiments were also used to evaluate the device performance of our Tm(IO_3_)_3_ single‐crystal detector in more detail. As shown in Figure 3f, the response times of [101] and [$\bar{1}01$] devices were 95.2 and 102.6 ms under a pulsed X‐ray (5.2 mGy_air_ s^−1^), as calculated from the duration between 10% and 90% photocurrent intensity. Thanks to the wide bandgap of Tm(IO_3_)_3_, no observable rise of the dose rate‐dependent photocurrents is found in Figure 3g under white light, demonstrating the favorable stability of the Tm(IO_3_)_3_ device in the presence of interference from visible light. Subsequently, we subjected the two single‐crystal devices to repeated X‐ray (5.2 mGy_air_ s^−1^) with 90 cycles and continuous X‐ray radiation (16.5 mGy_air_ s^−1^) with about 500 Gy_air_ in a high electric field of 233.8 V mm^−1^, as shown in Figure 3h,i; current traces devoid of any significant decline confirm that our devices are stable under the working conditions when subjected to frequent on–off and high‐dose radiation.

**Figure 4:**
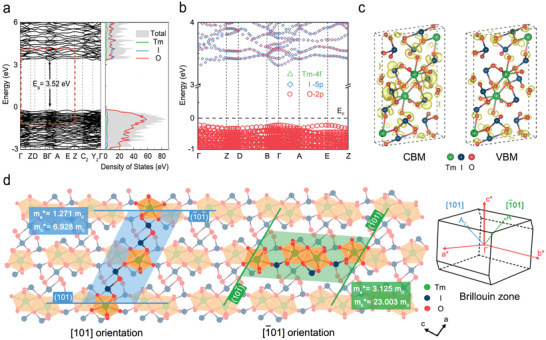
DFT calculation for Tm(IO_3_)_3_. a) Band structure and density of states. b) Orbital projected band structures. c) Charge distributions corresponding to the conduction band minimum (CBM) and valence band maximum (VBM). d) Schematic showing charge transport in a Tm(IO_3_)_3_ crystal in the two orientations. Inset: [101] and [$\bar{1}01$] orientations in the Brillouin zone.

As stated above, our X‐ray detector prepared using a Tm(IO_3_)_3_ single crystal exhibited an anisotropic response in X‐ray detection performance and outstanding operational stability; then we further discussed these in the electronic structure by density‐functional theory (DFT) calculations. In **Figure** **4**a, a theoretical bandgap of 3.52 eV was extracted from the direct band structure of Tm(IO_3_)_3_, which is acceptably close (difference of only 0.58 eV) to the experimental value, as well as I and O atoms contribute significantly to the band structure close to the Fermi level (*E*_F_). The working stability of a scintillation semiconductor is greatly affected by the bandgap value because a wide bandgap semiconductor is more radiation resistant than one with a narrow bandgap. Orbital contributions are also shown in Figure 4b; I‐5p and O‐2p states are almost equally dispersed in the conduction band near *E*_F_, while the O‐2p state mainly dominated the valence band near *E*_F_. It is worth noting that the valence band is more flatly dispersed than the conduction band, which is disadvantageous for the transporting p‐type carriers. In addition, the charge distributions of the valence band maximum (VBM) and the conduction band minimum (CBM) were investigated to trace the origin of the X‐ray detection performance shown by our detectors, the results of which are displayed in Figure 4c. It can be discovered that charges of I—O groups are observed in both the CBM and VBM as primary contributions, with less charge observed around the Tm atoms, indicating that I—O groups play a major role in charge transport of Tm(IO_3_)_3_. Subsequently, the paths in [101] and [$\bar{1}01$] orientations are depicted in Figure 4d, which represent the charge transport along the intra‐ and interlayer of Tm—I—O layers, respectively. The two paths have obviously different arrangements of the I—O and Tm—O groups; there are more I—O groups and fewer Tm—O groups in the [101] orientation than in the [$\bar{1}01$] orientation. Furthermore, the values of effective mass in the plane of CBM (electrons) and VBM (holes) were calculated to evaluate the transport properties of carriers along two [$\bar{1}01$] and [101] orientations, the Γ → A and Γ → A_2_ directions in the Brillouin zone (Figure 4d, inset). The calculated effective masses of electrons (m_e_*) in the [101] (1.271 *m*_0_) and the [$\bar{1}01$] (3.125 *m*_0_) orientations are much smaller than that of holes (m_h_*) in the corresponding orientation (6.928 and 23.003 *m*_0_, respectively), which demonstrates that electrons dominate carrier transport, while holes are more difficult to move in each orientation due to a more flatly dispersed valence band. Notably, the [101] orientation with smaller m_e_* has greater conductivity and larger sensitivity than the [$\bar{1}01$] orientation, as well as a lower detection limit is exhibited in the [$\bar{1}01$] orientation because of its higher resistivity and less ion migration that endows it with a low dark current and little baseline drifting. Based on the analysis of anisotropy for material properties and X‐ray detection performances, we conclude that the I—O groups favor electrical conduction while Tm—O groups act as spacers that limit charge transport between I—O groups; hence, different arrangements of these groups are responsible for the anisotropy of electronic property in Tm(IO_3_)_3_.

**Figure 5:**
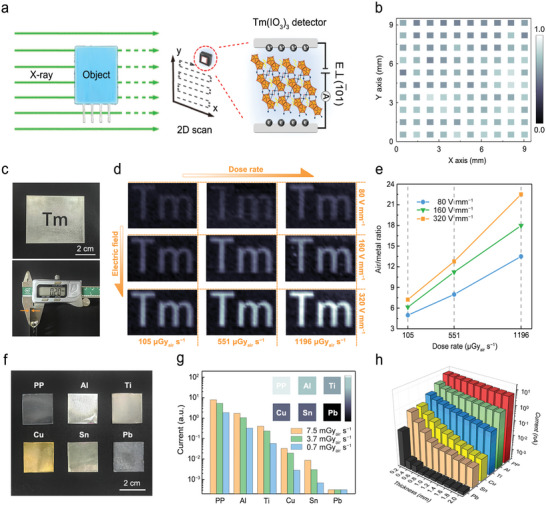
Imaging performance of the Tm(IO_3_)_3_ device. a) Scheme of the single‐pixel 2D‐scan X‐ray imaging system. b) Photocurrent response of the Tm(IO_3_)_3_ device over a 10 × 10 mm^2^ scan area. c) Photographic image (upper) and thickness measurement (lower) of stainless steel plate. d) X‐ray images of stainless steel plates in various electric fields and at different dose rates. e) Air/metal intensity ratio as functions of electric field and dose rate. f) Photographic images of materials with various densities. g) Current intensity comparison for 1.0‐mm‐thickness materials at various dose rates. Inset: X‐ray images of materials with various densities. h) Current intensity comparison for materials with different thicknesses.

A high‐performance, durable X‐ray imaging device with low working doses is highly desired for practical applications. To illustrate the feasibility of Tm(IO_3_)_3_ crystal in X‐ray imaging applications, we built a single‐pixel 2D‐scan imaging platform based on the [$\bar{1}01$] orientation device (with low detection limit and great operational stability), schematically displayed in **Figure** **5**a.

Uniform scanning responses are required during X‐ray imaging; consequently, we evaluated signal uniformity by examining the detector in a 10 × 10 mm^2^ area when exposed to 1196 µGy_air_ s^−1^ X‐ray radiation at a constant scan rate of 1 mm s^−1^, as shown in Figure 5b. No obvious variation in photocurrent was observed, which indicates that the detector responds stably during motion (Figure S21, Supporting Information).

We examined the electric field and dose rate, which are two leading factors influencing direct‐type X‐ray imaging performance, in order to optimize the working conditions for the X‐ray imaging system. As shown in Figure 5c,d, a hollowed‐out 0.2‐mm‐thickness stainless steel letter “Tm” was used as a testing object in imaging experiments in which various electric fields (80–320 V mm^−1^) and dose rates (105–1196 µGy_air_ s^−1^) were applied. Encouragingly, higher dose rates and electric fields provided better X‐ray images, with optimal imaging performance observed at 320 V mm^−1^ and 1196 µGy_air_ s^−1^; the horizontal contour profiles of the “Tm” image (red line) supply further details (Figure S22, Supporting Information). It should be noted that the difference in X‐ray responses between air and metal is further enhanced in higher electric fields and at larger dose rates, leading to a higher air/metal intensity ratio (Figure 5e), which is mainly ascribable to the high resistivity and low current drifting of the [$\bar{1}01$] device, preventing sharp rises in dark current under these conditions.

In addition, the capacity to distinguish between materials with various densities was also assessed using five metals (Pb, Cu, Sn, Ti, and Al) and polypropylene (PP) as 1.0‐mm‐thickness attenuation plates (Figure 5f); the imaging colors of these materials are clearly differentiable in the X‐ray image (Figure 5g), as also confirmed by the current intensities observed for these materials at three dose rates (0.7–7.5 mGy_air_ s^−1^). The constant intensity for Pb at various dose rates indicates that the Pb plate (1 mm) most completely attenuates 50 keV X‐ray. The density resolution capability was further studied using different materials with various thicknesses (Figure 5h), which demonstrated that our device can fundamentally be used in high‐performance imaging applications.

**Figure 6:**
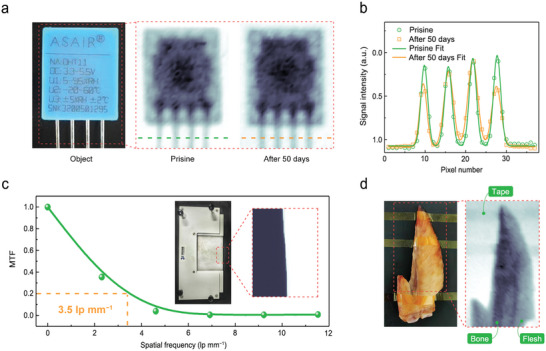
Imaging applications of the Tm(IO_3_)_3_ device. a) Optical image (left) and X‐ray images (right) of the sensor before and after 50 days. b) Contour profiles of the X‐ray image. c) Modulation transfer function (MTF) of our Tm(IO_3_)_3_ device as a function of spatial frequency. Inset: optical (left) and X‐ray (right) image of tungsten edge. d) Optical (left) and X‐ray (right) image of a chicken wing.

Several X‐ray detector performances that determine applicable feasibility were investigated using the Tm(IO_3_)_3_ device. As shown in **Figure** **6**a, The plastic shell and metal components of the sensor are clearly visible in X‐ray images before and after 50 days, and the Gaussian fitting of a four‐metal‐pillar profile shows peaks with uniformly distributed intensities (Figure 6b); the contrast of metal pillars degraded slightly (by 8%) over 50 days in these X‐ray images, which is indicative of outstandingly stable imaging performance. Such stable performance is attributed to the excellent material stability of Tm(IO_3_)_3_ (Figure S23, Supporting Information). According to the slanted‐edge method, the spatial resolution of the Tm(IO_3_)_3_ X‐ray detector was determined to be 3.5 lp mm^−1^ in the imaging system (Figure 6c); the black‐white image was obtained by a 1.0‐mm‐thickness tungsten plate to calculate the edge spread function and the line spread function (Figure S24, Supporting Information). Furthermore, objects imaged in medical applications have more demanding requirements due to complex components that include bone, flesh, plastic and metal, as examples. Obviously, the outline of a chicken wing and its bone/flesh boundary are observed in Figure 6d; 60‐µm‐thickness polyimide tapes are also distinguished, demonstrating a discernible dose rate: ≈17 µGy_air_ s^−1^, which was calculated using the equation: *I* = *I*_0_∙e^−µt^, where *I*_0_ is the initial dose rate, µ is the linear attenuation coefficient and *t* is the thickness of materials. These results confirm the significant potential of our iodate device in actual imaging applications.

## 3 Conclusion

We developed a rare‐earth iodate Tm(IO_3_)_3_ single crystal as a novel scintillation semiconductor with an ultra‐wide bandgap of 4.1 eV, for high‐performance direct X‐ray detection applications. Anisotropic responses along [101] and [$\bar{1}01$] crystal orientations were exhibited in material properties and detection performances of Tm(IO_3_)_3_ single‐crystal devices; an extremely low dark current density (0.6 nA cm^−2^) and small baseline drifting (0.2 × 10^−2^ nA cm^−1^ s^−1^ V^−1^) obtained in [$\bar{1}01$] crystal orientation leads to the low detection limit of 85.2 nGy_air_ s^−1^. Moreover, high sensitivity of 4406.6 µC Gy_air_^−1^ cm^−2^ and superior operation stability under 507.5 Gy_air_ were achieved in the [101] device with a vertical structure. The anisotropy is attributed to structural differences between the two orientations in Tm(IO_3_)_3_, which indicates that X‐ray detection is affected by the arrangement of the I—O and Tm—O groups. Tm(IO_3_)_3_ was subjected to DFT calculations to compare the effective masses of the carriers along two orientations and to verify the relationship between structure and anisotropic performances. In addition, the feasible use of our detector in high‐quality X‐ray imaging applications was confirmed using a self‐built 2D‐scan imaging system. This work provides a novel perspective for the structure design of iodate semiconductors that involves group arrangements, and is expected to inspire the exploration of novel materials for next‐generation X‐ray imaging systems.

## 4 Experimental Section

### Crystal Growth and Detector Fabrication

Sodium iodate (NaIO_3_; 99%, Aladdin Reagent Ltd.) and thulium nitrate pentahydrate (Tm(NO_3_)_3_·5H_2_O; 99.99%, Shanghai Diyang Industrial Co., Ltd.) were dissolved in 30 mL deionized water (Aladdin Reagent Ltd.) at a 1:0.24 mass ratio, as well as 1 mL Nitric acid (HNO_3_; AR, Aladdin Reagent Ltd.) was added to promote crystal growth. The mixture was added to a Teflon‐lined stainless steel autoclave (50 mL) and heated at a constant temperature of 200 °C for 120 h, and then cooled with three programmed segments (200 to 150 °C, 150 to 100 °C, and 100 to 20 °C) at different rates of 0.1 to 1 °C h^−1^ in a heating oven. Light yellow millimeter‐sized Tm(IO_3_)_3_ crystals were washed with deionized water and obtained by filtration. The CdTe single crystals were grown along with [111] crystal orientation and polished on one side, purchased from Hefei Kejing Materials Technology Co., Ltd. On the top and bottom surfaces of the Tm(IO_3_)_3_ single crystals along two crystal orientations, silver electrodes (1.0 Troy oz., Structure Probe. Inc.) were produced, and gold bonding wire (JC204C, JoBo) was bonded to the silver electrodes as conductive testing wire. The CdTe detectors were produced by the same process.

### Material Characterization

Power XRD patterns were collected using Cu K*α* radiation (40 kV, 15 mA) with 0.01° steps on a diffractometer (Rigaku Miniflex600). Rocking curves were acquired by a Rigaku SmartLab instrument with Cu K*α* X‐rays (40 kV, 30 mA). Crystal structure and crystalline orientation were obtained on a Synergy Custom (Liquid MetalJet D2+). UV–Visible absorption spectra were produced on a spectrometer (Hitachi U4100UV). Thermogravimetric analyses were acquired on a thermal gravimetric analyzer (Setaram Setsys16) under continuous nitrogen flow. The temperature–conductivity curves were conducted on a Novocontrol Technologies broadband dielectric/impedance spectrometer at a bias of 1 V. Electrode area and crystal thickness were measured using a video measuring system (JTVMS‐3020, Dongguan Jaten Precision Instrument Co., Ltd.). Optical images were acquired using a cell phone (HUAWEI P50 pro).

### DFT Calculation

The first‐principles calculations were carried out by the Vienna ab initio simulation package^[^ ^46^ ^]^ and the electron–ion interaction was described using the projector augmented wave method.^[^ ^47^ ^]^ Perdew–Burke–Ernzerhof and generalized gradient approximation were performed to relax the structural configurations and calculate the band structure.^[^ ^48^ ^]^ A plane‐wave energy cutoff of 500 eV was used in all calculations. All structures were geometrically relaxed until the total force on each ion was below 0.01 eV Å^−1^. The K‐point pathway was defined as follows: Γ(0 0 0), Z(0 0.5 0), D(0 0.5 0.5), B(0 0 0.5), A(−0.5 0 0.5), E(−0.5 0.5 0.5), C_2_(−0.5 0.5 0), and Y_2_(−0.5 0 0). The paths along [$\bar{1}01$] and [101] orientations, Γ (0 0 0) → A (−0.5 0 0.5) and Γ (0 0 0) → A_2_ (0.4 0 0.4), were set to calculate effective mass using the VASP processing program VASPKIT.^[^ ^49^ ^]^

### X‐Ray Detection and X‐Ray Imaging

A tungsten anode X‐ray tube (Moxtek TUB00154‐W06) with 50 kV voltage and a tungsten anode X‐ray tube (Hamamatsu L9181‐02) with 50–120 kV voltage were used to provide a continuous X‐ray, and a self‐built chopper manufactured by lead sheets of 0.5 mm was used to produce a pulsed X‐ray beam. A dosimeter (Radical AGDM+) was used to calibrate X‐ray dose rates, and 1, 2 and 10 mm Aluminum plates as attenuators were employed to achieve a low dose rate. To provide the bias voltage and record current data, a computer‐controlled source meter (Keithley Model 2450) with a test fixture (Keithley Model 8101‐PIV) was employed. Dark noise current was collected using a low‐noise current preamplifier (Stanford Research System, SR570) and a digital oscilloscope (Rigol, DS1052E) at 5 V. A home‐built 2D‐scan imaging system, including an X‐ray tube, a 2D mobile platform, an X‐ray detector built of a Tm(IO_3_)_3_ single crystal and a data‐gathered Keithley 2450 source meter, was used for X‐ray imaging. A 1.0‐mm‐thick tungsten edge (IEC 62220‐1‐3:2008) was used in this work. Imaging objects were bought from the online store of Taobao.com. All X‐ray experiments were performed in a dark, closed room (40–55% relative humidity) that was controlled from outside the room by the researcher.

CCDC 2213863 contains supplementary crystallographic data for this paper. These data can be obtained free of charge from The Cambridge Crystallographic Data Centre via www.ccdc.cam.ac.uk/data_request/cif.

## Conflict of Interest

The authors declare no conflict of interest.

## Author Contributions

S.W. and S.F.W. conceived and supervised the project. X.X. performed most of the experiments in this work. F.W. assisted with X‐ray detection and X‐ray imaging. W.X. assisted with crystal growth. H.L. assisted with detector fabrication and X‐ray tube calibration. L.L. assisted with the temperature‐dependent conductivity and frequency‐dependent dielectric constant tests. H.S. assisted with the birefringence test and its analysis. X.J. assisted with DFT calculations. S.W. and X.X. wrote the manuscript and all authors reviewed it.

## Supporting information

Supporting Information

## Data Availability

Research data are not shared.
