# Supplementary material for: Wide‐Bandgap Rare‐Earth Iodate Single Crystals for Superior X‐Ray Detection and Imaging
Source: Adv Sci (Weinh). 2023 Mar 22;10(14):2206833. doi: 10.1002/advs.202206833 (PMC10190661; doi:10.1002/advs.202206833)
Supplement: Supplementary file 1 — Supporting Information [file ADVS-10-2206833-s001.pdf]

Supporting Information

Wide-Bandgap Rare-Earth Iodate Single Crystals for Superior X-Ray Detection and Imaging

*Xieming Xu[1,3], Fang Wang[1], Weiwei Xu[1], Hao Lu[1,3], Lingfei Lv[1,3], Hongyuan Sha[1,3], Xiaoming Jiang[1], Shaofan Wu[1], and Shuaihua Wang[1,2]**

[1] Key Laboratory of Optoelectronic Materials Chemistry and Physics, Fujian Institute of Research on the Structure of Matter, Chinese Academy of Sciences, Fuzhou, Fujian 350002, China.

[2] Fujian Science & Technology Innovation Laboratory for Optoelectronic Information of China, Fuzhou, Fujian 350002, China.

[3] University of Chinese Academy of Sciences, Beijing 100049, China.

* e-mail: shwang@fjirsm.ac.cn.

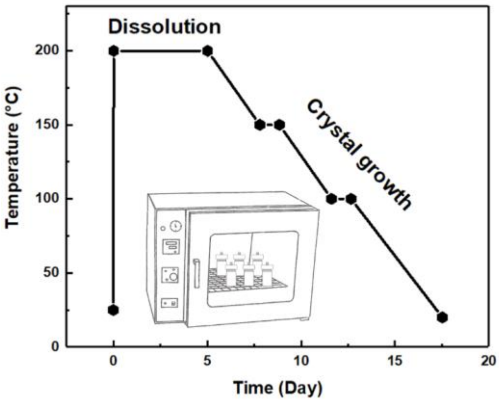

Figure S1 Temperature-ramp profile of the hydrothermal reaction for crystal growth.

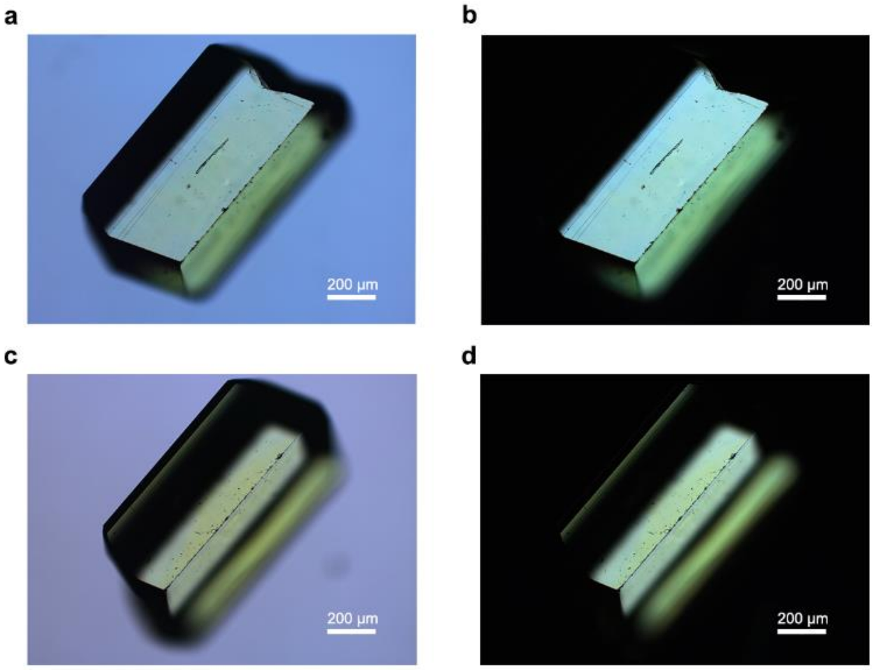

Figure S2 Optical microscopy images of $Tm(IO_3)_3$ SC in (a) normal mode of $(\bar{1}01)$ face, (b) polarized mode of $(\bar{1}01)$ face, (c) normal mode of (101) face and (d) polarized mode of (101) face, respectively.

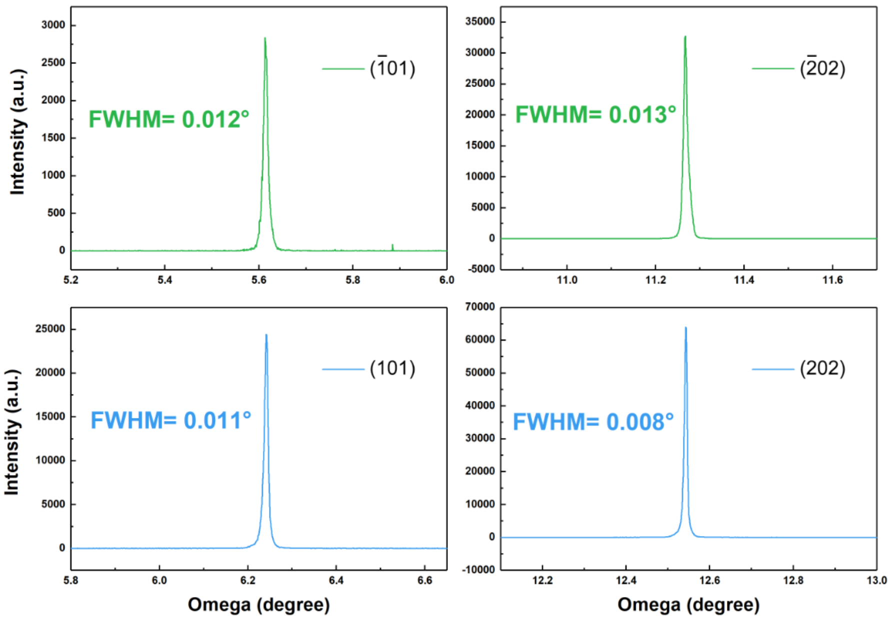

Figure S3 Rocking curve of different crystalline faces: a) $(\bar{1}01)$ face, b) $(\bar{2}02)$ face, c) (101) face, and d) (202) face of a high-quality $Tm(IO_3)_3$ single crystal.

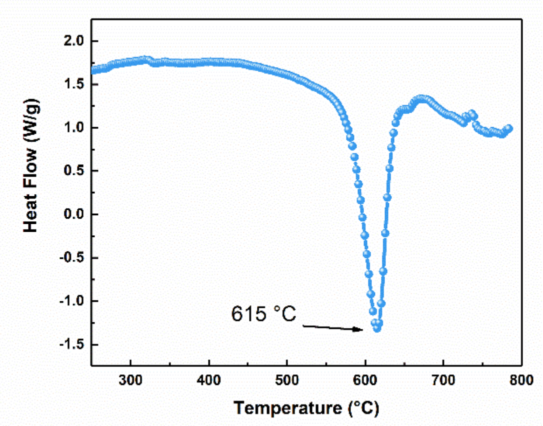

Figure S4 The thermal performance of the $Tm(IO_3)_3$ crystal measured using differential scanning calorimetry (DSC) analyses.

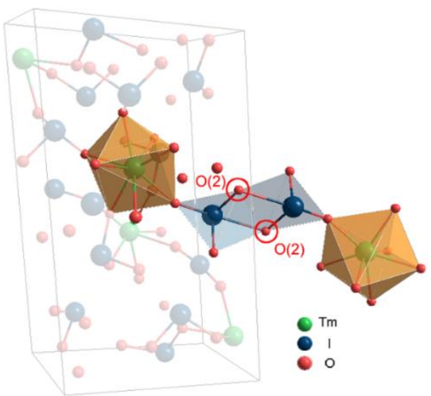

Figure S5 Symmetric transformation of IO_{3+1} polyhedron in the $Tm(IO_3)_3$.

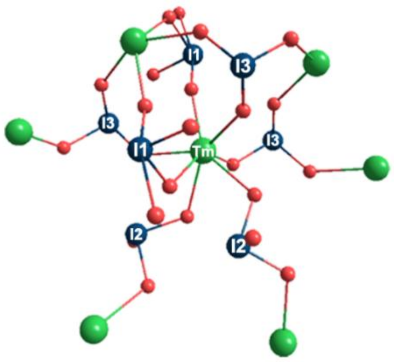

Figure S6 Coordination environment in the $Tm(IO_3)_3$.

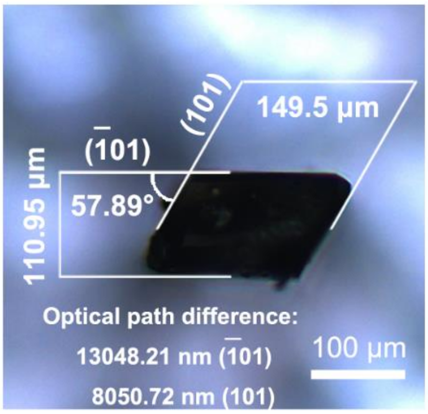

Figure S7 The shape parameters of the $Tm(IO_3)_3$ single crystal.

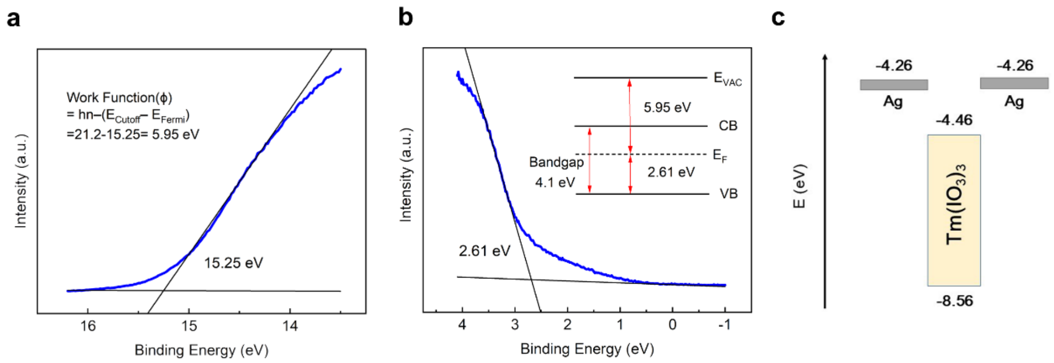

Figure S8 a) and b) The UPS spectrum of the $Tm(IO_3)_3$ powder. c) the energy level diagram of $Tm(IO_3)_3$ device.

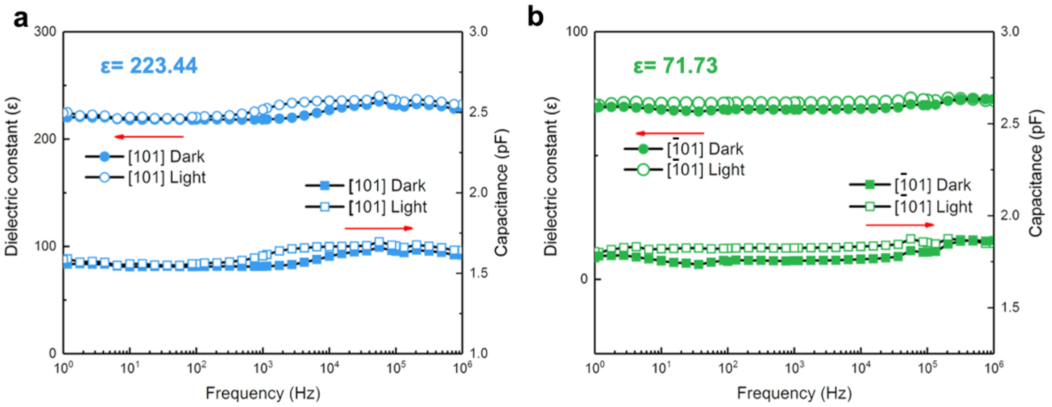

Figure S9 Frequency-dependent capacitances and dielectric constant curves in the dark and light of $Tm(IO_3)_3$ single crystal along a) [101] and [$\bar{1}01$] orientations.

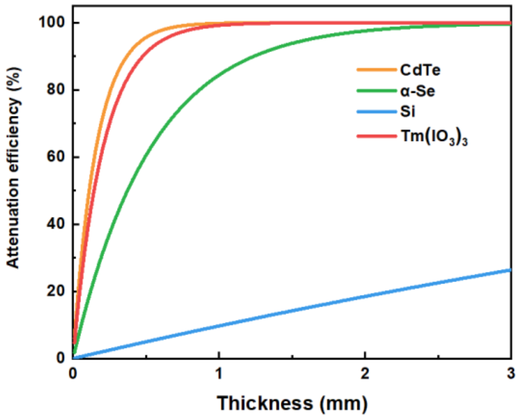

Figure S10 Thickness-dependent absorption efficiency of Si, α-Se, CdTe, and $Tm(IO_3)_3$.

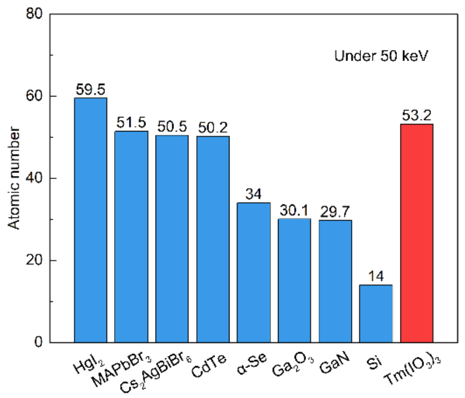

Figure S11 Comparison of the atomic number for various scintillation materials.

The effective atomic number of the compound was calculated by the direct method[1], according to the equation: $Z_{eff} = \frac{\sum f A \mu_m}{\sum f A \mu_m / Z}$, where f is the molar fraction in the compound, A is the atomic weight, μ_m is the mass attenuation coefficient under one energy (50 keV), and Z is the atomic number. The value of μ_m can be obtained from XCOM: Photon cross sections database (https://physics.nist.gov/PhysRefData/Xcom/html/xcom1.html).

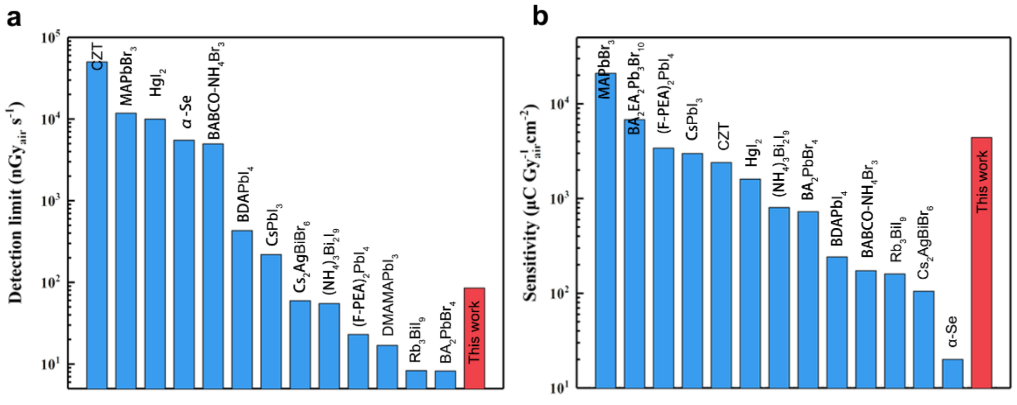

Figure S12 Comparison of a) detection limit and b) sensitivity between various scintillation materials.

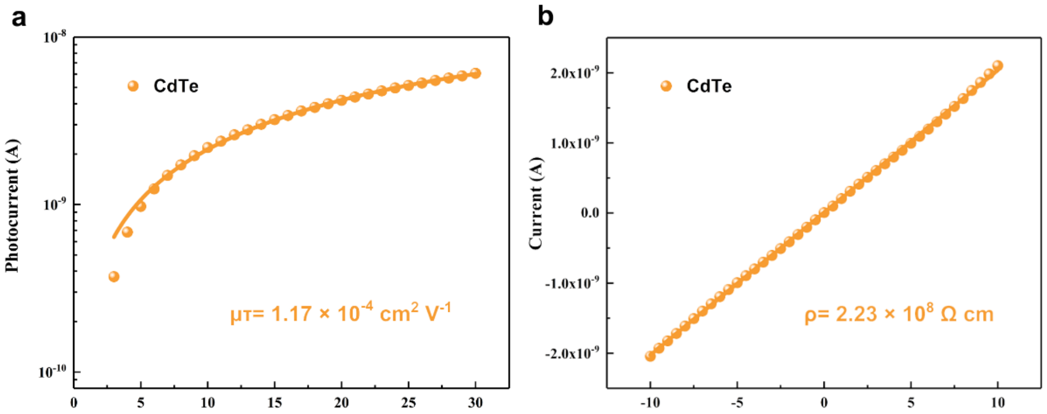

Figure S13 a) Bias-dependent photoconductivity measurement of CdTe. b) I-V curve of CdTe in the dark.

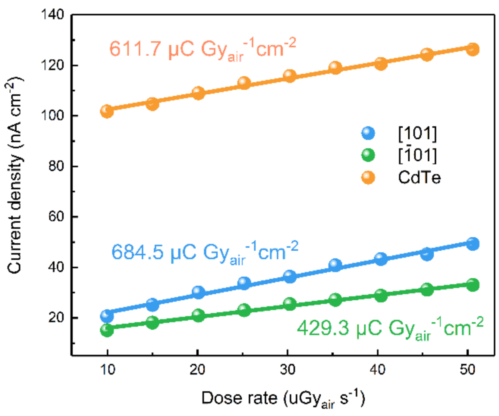

Figure S14 Dose rate-dependent X-ray current density for the [101] and [$\bar{1}$01] devices of $Tm(IO_3)_3$ and CdTe device at 11.7 V mm^{-1}.

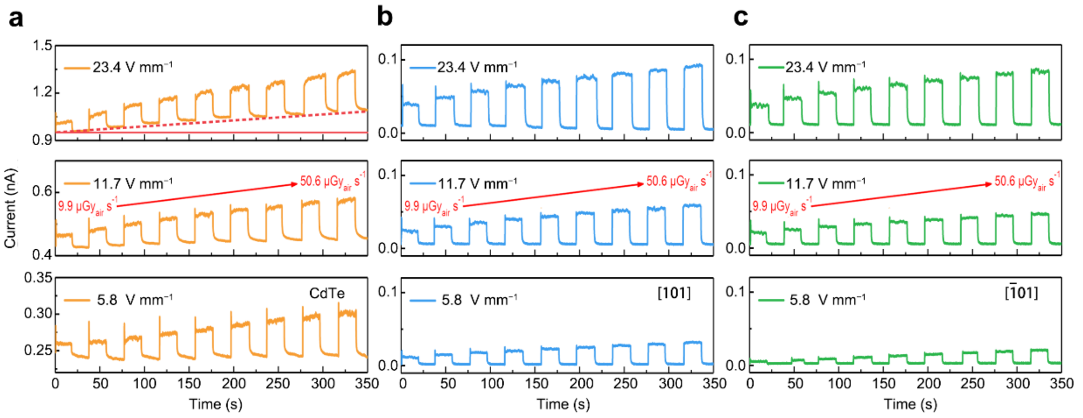

Figure S15 Photocurrent response of a) the CdTe device, b) the [101] device of $Tm(IO_3)_3$, and c) the [$\bar{1}$01] device of $Tm(IO_3)_3$ with various dose rate (9.9–50.6 $\mu Gy_{air}\ s^{-1}$) under three electric fields.

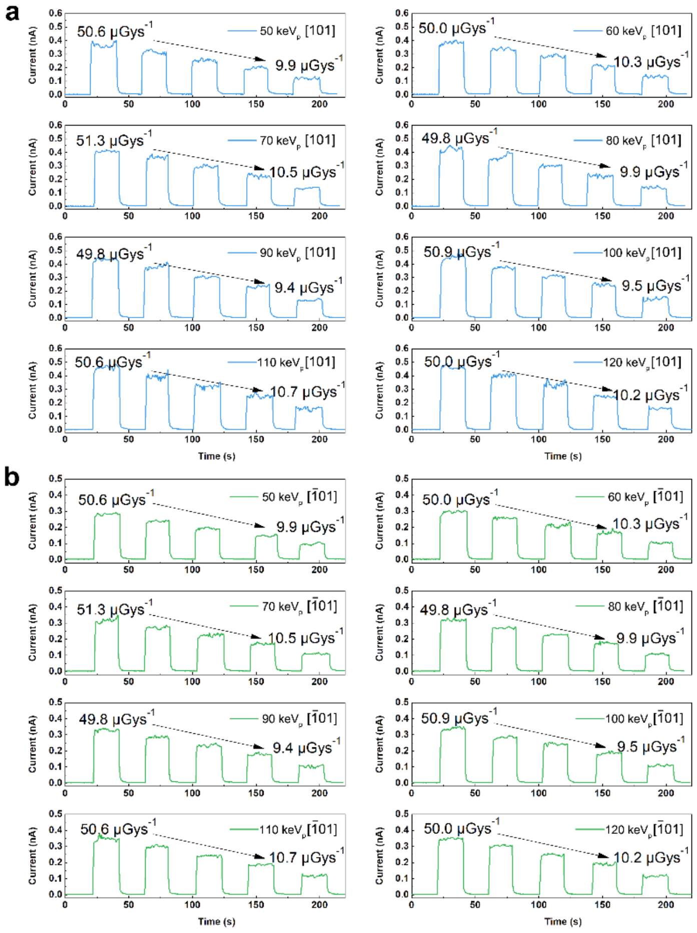

Figure S16 Photocurrent response of a) the [101] device of $Tm(IO_3)_3$ and b) the [$\bar{1}$01] device of $Tm(IO_3)_3$ with different X-ray energies (50-120 keV_p) at the dose rate region of 10–50 $\mu Gy_{air}\ s^{-1}$ under 233 V mm^{-1} electric fields.

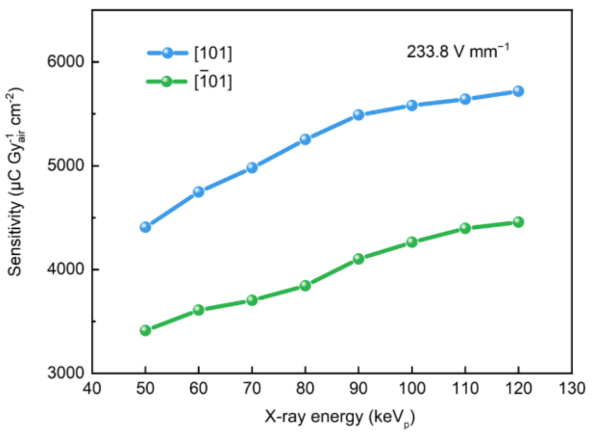

Figure S17 The X-ray energy-dependent sensitivity for the [101] and [$\bar{1}$01] devices of $Tm(IO_3)_3$ at 233.8 V mm^{-1}.

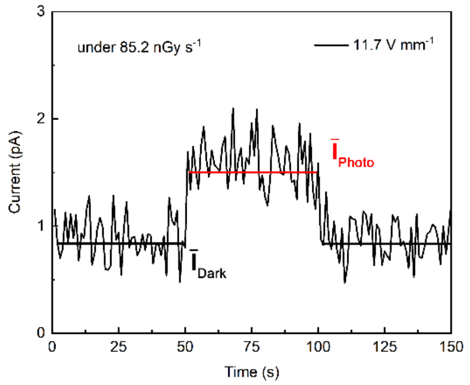

Figure S18 The temporal response under the X-ray dose rate of 85.2 nGy$_{air}$ s^{-1}.

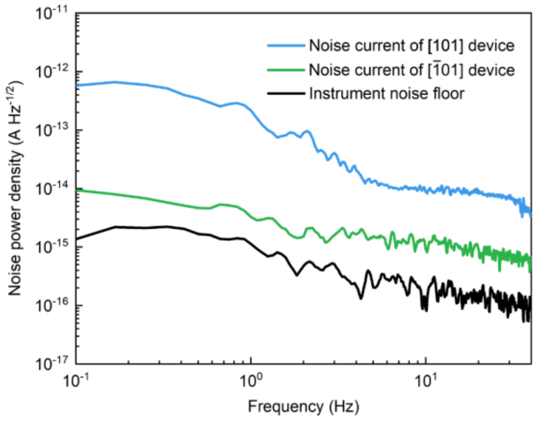

Figure S19 Measured dark noise current and instrument noise at various frequencies

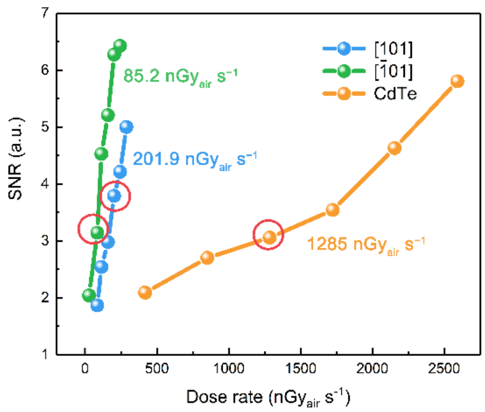

Figure S20 X-ray dose rate-dependent signal-to-noise ratio (SNR) of the CdTe and two $Tm(IO_3)_3$ devices at 11.7 V mm^{-1}.

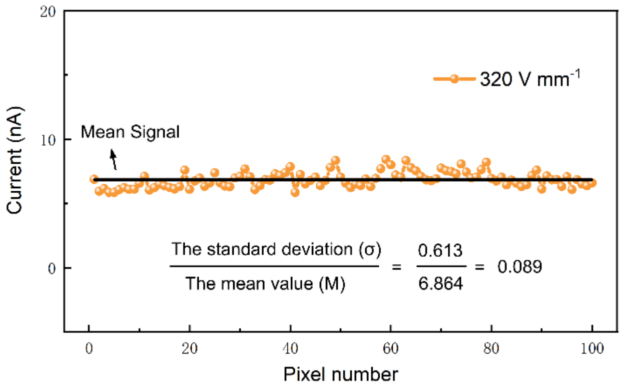

Figure S21 X-ray response distribution curve acquired during stability testing.

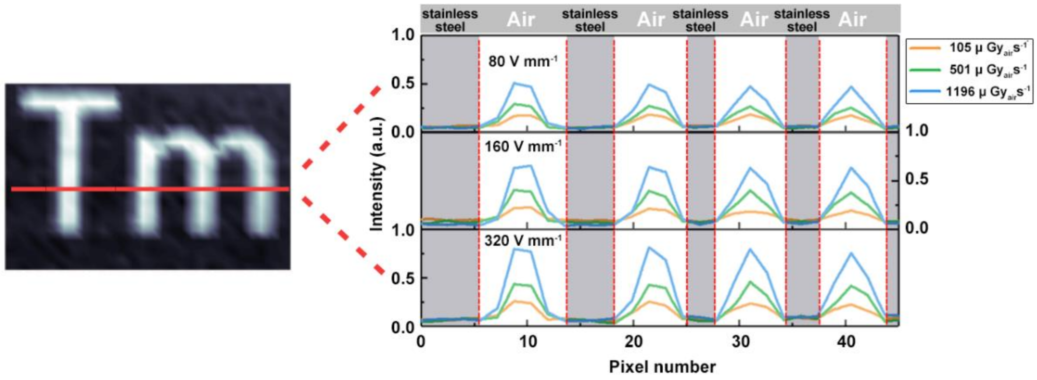

Figure S22 The horizontal contour profiles of the “Tm” letter X-ray image.

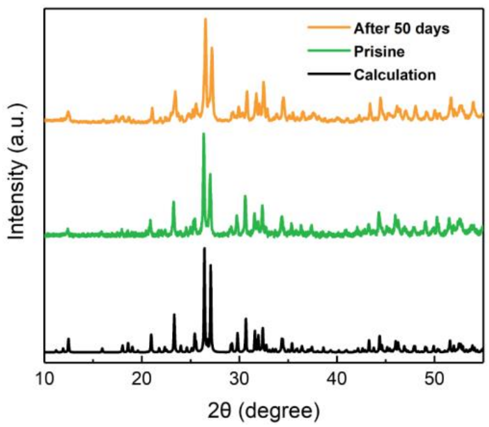

Figure S23 Prisine and after 50 days of the $Tm(IO_3)_3$ XRD patterns.

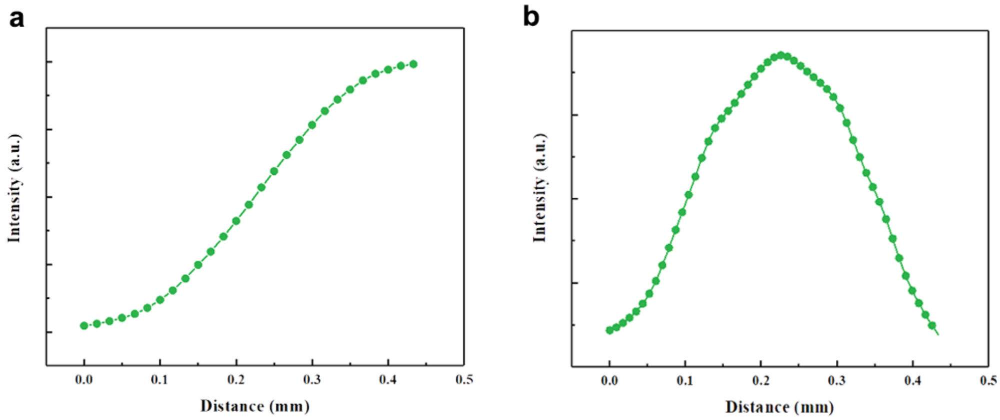

Figure S24 X-ray response current for the edge of the tungsten plate measured as the edge spread function (ESF) and the line spread function (LSF).

Table S1. Crystal data and structural refinement for $Tm(IO_3)_3$.

**Formula**	**$Tm(IO_3)_3$**
Formula weight	693.63
Temperature (K)	293.2
λ(Å)	1.3405
Crystal system	Monoclinic
Space group	$P2_1/n$
a (Å)	8.6897 (2)
b(Å)	5.99050 (10)
c (Å)	14.9324 (2)
α (deg)	90
β (deg)	96.960 (2)
γ (deg)	90
V ($Å^3$)	771.59 (2)
Z	4
ρ(calcd) (g/cm^3)	5.971
μ (mm^{-1})	102.797
F(000)	1200
θ (deg)	4.878- 53.848
Index range	$-10 \le h \le 10$<br>$-7 \le k \le 3$<br>$-17 \le l \le 17$
Reflections collected / unique	4518 / 1408
R_{int}	0.0733
Completeness to θ = 53.543° (%)	99.9
GOF on F^2	1.063
R_1/wR_2 $[F_o^2 > 2\sigma(F_o^2)]^a$	0.0489 / 0.1240
R_1/wR_2 (all data)	0.0507 / 0.1261
Extinction coefficient	0.00039 (9)

[a] $R_1(F) = \sum||F_o| - |F_c||/\sum|F_o|$; $wR_2(F_o^2) = [\sum w(F_o^2 - F_c^2)^2/\sum w(F_o^2)^2]^{1/2}$

Table S2. Selected bond lengths (Å) for $Tm(IO_3)_3$.

Tm(1)-O(3)#1	2.352(9)	I(1)-O(4)	1.815(8)
Tm(1)-O(4)#1	2.331(9)	I(1)-O(8)	1.843(9)
Tm(1)-O(5)	2.293(8)	I(2)-O(1)	1.805(9)
Tm(1)-O(6)#2	2.360(8)	I(2)-O(6)	1.812(8)
Tm(1)-O(7)#3	2.293(9)	I(2)-O(9)	1.822(9)
Tm(1)-O(8)	2.237(9)	I(3)-O(3)	1.811(9)
Tm(1)-O(9)	2.357(9)	I(3)-O(5)	1.789(8)
I(1)-O(2)#4	2.457(10)	I(3)-O(7)	1.812(9)
I(1)-O(2)	1.832(9)		

Symmetry transformations used to generate equivalent atoms:
#1 -x+1,-y,-z+1 #2 -x+3/2,y-1/2,-z+1/2
#3 -x+1,-y+1,-z+1 #4 -x,-y,-z+1

Table S3. Bond Valence Sums (BVS) Calculations for $Tm(IO_3)_3$.

**Tm(1)**		**I(1)**		**I(2)**		**I(3)**	
**Group**	**BVS**	**Group**	**BVS**	**Group**	**BVS**	**Group**	**BVS**
TmO_7	2.98647	IO_{3+1}	5.04239	IO_3	4.97574	IO_3	5.09976
TmO_8	3.09413	IO_3	4.75155				
TmO_9	3.13094						

Table S4. Material parameters of scintillation semiconductors.

Materials	Bandgap (eV)	Effective Atomic Number[a]	Density ($g\ cm^{-3}$)	μτ product ($cm^{-2}\ V^{-1}$)	Resistivity (Ω cm)	Refs.
$MAPbBr_3$	2.23	51.5	3.78[b]	10^{-2}	1.25×10^{8}	[2]
BA_2PbBr_4	2.94	43.0	2.38[b]	1.1×10^{-5}	10^{11}–10^{12}	[3]
$Cs_2AgBiBr_6$	2.10	50.5	4.93[b]	6.3×10^{-3}	10^{9}–10^{11}	[4]
$CsPbI_3$	2.67	57.6	5.02[b]	3.63×10^{-3}	2.82×10^{7}	[5]
InSe	2.10	44.9	5.24	5×10^{-4}	1.1×10^{11}	[6]
GaN	3.39	29.7	6.15	—	$>10^{6}$	[7,8]
β-Ga_2O_3:Mg	4.70	～30.1	～6.44	—	6.4×10^{11}	[9]
Si	1.12	14.0	2.33	>1	10^{4}	[10-12]
α-Se	2.25	34.0	4.3	10^{-7}	10^{14}–10^{15}	[13]
HgI_2	2.13	59.5	6.4	10^{-4}	10^{13}	[14]
$Cd_{0.9}Zn_{0.1}Te$	1.57	50.0	5.78	10^{-2}	10^{11}	[15]
CdTe	1.50	50.2	6.2[b]	1.17×10^{-4}	2.23×10^{8}	This work, [16]
**$Tm(IO_3)_3$**	**4.10**	**53.2**	**5.97[b]**	**6.61×10^{-5}** **1.35×10^{-4}**	**2.27×10^{10}** **1.02×10^{11}**	**This work**

a. The effective atomic number values were calculated by the direct method[1] in 50 keV.
b. The bandgap values were obtained from the crystal structure data.

Table S5. Detection performances of direct X-ray detectors.

Materials	X-ray energy (keV_p)	Detection limit ($\mu Gy_{air}\ s^{-1}$)	Sensitivity ($\mu C\ Gy_{air}^{-1} cm^{-2}$)	Electric field ($V\ mm^{-1}$)	Refs.
$MAPbBr_3$	8	11.8	21000	N/A	[2]
α-Se	20	5.5	20	10000	[13]
$BDAPbI_4$	40	0.43	242	310	[17]
BA_2PbBr_4	50	0.0082	726.18	−920	[3]
$Cs_2AgBiBr_6$	50	0.0596	105	25	[4]
β-Ga2O3:Mg	50	69.5	338.9	−1000	[9]
HgI_2	70	10	1600	250	[14]
$Cd_{0.9}Zn_{0.1}Te$	80	50	2400	250	[15]
InSe	80	5.35	3.96	N/A	[6]
$CsPbI_3$	80	0.0219	2990	30	[5]
CdTe	50	1.29	611.7	11.7	This work
**$Tm(IO_3)_3$**	**50**	**0.085**	**4406.6**	**233.8**	**This work**

References

[1] S. R. Manohara, S. M. Hanagodimath, K. S. Thind, L. Gerward, *Nucl. Instrum. Methods Phys. Res., Sect. B* **2008**, *266*, 3906.

[2] W. Wei, Y. Zhang, Q. Xu, H. Wei, Y. Fang, Q. Wang, Y. Deng, T. Li, A. Gruverman, L. Cao, J. Huang, *Nat. Photon.* **2017**, *11*, 315.

[3] X. M. Xu, Y. H. Wu, Y. Zhang, X. H. Li, F. Wang, X. M. Jiang, S. F. Wu, S. H. Wang, *Energy Environ. Mater.***2022**, e12487.

[4] W. C. Pan, H. D. Wu, J. J. Luo, Z. Z. Deng, C. Ge, C. Chen, X. W. Jiang, W. J. Yin, G. D. Niu, L. J. Zhu, L. X. Yin, Y. Zhou, Q. G. Xie, X. X. Ke, M. L. Sui, J. Tang, *Nat. Photon.* **2017**, *11*, 726.

[5] B.-B. Zhang, X. Liu, B. Xiao, A. Ben Hafsia, K. Gao, Y. Xu, J. Zhou, Y. Chen, *J. Phys. Chem. Lett.* **2020**, *11*, 432.

[6] S. J. Wu, C. Y. Liang, J. X. Zhang, Z. Wu, X. L. Wang, R. Zhou, Y. X. Wang, S. A. Wang, D. S. Li, T. Wu, *Angew. Chem., Int. Ed.* **2020**, *59*, 18605.

[7] M. Sato, *Appl. Phys. Lett.* **1996**, *68*, 935.

[8] S. J. Pearton, J. C. Zolper, R. J. Shul, F. Ren, *J. Appl. Phys.* **1999**, *86*, 1.

[9] J. W. Chen, H. L. Tang, B. Liu, Z. C. Zhu, M. Gu, Z. X. Zhang, Q. Xu, J. Xu, L. D. Zhou, L. Chen, X. P. Ouyang, *ACS Appl. Mater. Interfaces* **2021**, *13*, 2879.

[10] G. Rikner, E. Grusell, *Phys. Med. Biol.* **1983**, *28*, 1261.

[11] M. Guerra, M. Manso, S. Longelin, S. Pessanha, M. L. Carvalho, *J. Instrum.* **2012**, *7*.

[12] Z. P. Luo, J. G. Moch, S. S. Johnson, C. C. Chen, *Curr. Nanosci.* **2017**, *13*, 364.

[13] E. Samei, M. J. Flynn, D. A. Reimann, *Med. Phys.* **1998**, *25*, 102.

[14] P. Buechele, M. Richter, S. F. Tedde, G. J. Matt, G. N. Ankah, R. Fischer, M. Biele, W. Metzger, S. Lilliu, O. Bikondoa, J. E. Macdonald, C. J. Brabec, T. Kraus, U. Lemmer, O. Schmidt, *Nat. Photon.* **2015**, *9*, 843.

[15] S. Tokuda, H. Kishihara, S. Adachi, T. Sato, *J. Mater. Sci.: Mater. Electron.* **2004**, *15*, 1.

[16] A. Owens, *Journal Of Synchrotron Radiation* **2006**, *13*, 143.

[17] Y. Shen, Y. C. Liu, H. C. Ye, Y. T. Zheng, Q. Wei, Y. D. Xia, Y. H. Chen, K. Zhao, W. Huang, S. Z. Liu, *Angew. Chem., Int. Ed.* **2020**, *59*, 14896.
